# BTC-fCNN: Fast Convolution Neural Network for Multi-class Brain Tumor Classification

**DOI:** 10.1007/s13755-022-00203-w

**Published:** 2023-01-02

**Authors:** Basant S. Abd El-Wahab, Mohamed E. Nasr, Salah Khamis, Amira S. Ashour

**Affiliations:** Department of Electronics and Electrical Communications Engineering, Faculty of Engineering, Tanta University, Tanta, Egypt

**Keywords:** Brain tumor classification, Convolution neural network, Average pooling layer, Convolution layer, Transfer learning

## Abstract

Timely prognosis of brain tumors has a crucial role for powerful healthcare of remedy-making plans. Manual classification of the brain tumors in magnetic resonance imaging (MRI) images is a challenging task, which relies on the experienced radiologists to identify and classify the brain tumor. Automated classification of different brain tumors is significant based on designing computer-aided diagnosis (CAD) systems. Existing classification methods suffer from unsatisfactory performance and/or large computational cost/ time. This paper proposed a fast and efficient classification process, called BTC-fCNN, which is a deep learning-based system to distinguish between different views of three brain tumor types, namely meningioma, glioma, and pituitary tumors. The proposed system’s model was applied on MRI images from the Figshare dataset. It consists of 13 layers with few trainable parameters involving convolution layer, 1 × 1 convolution layer, average pooling, fully connected layer, and softmax layer. Five iterations including transfer learning and five-fold cross-validation for retraining are considered to increase the proposed model performance. The proposed model achieved 98.63% average accuracy, using five iterations with transfer learning, and 98.86% using retrained five-fold cross-validation (internal transfer learning between the folds). Various evaluation metrics were measured to evaluate the proposed model, such as precision, F-score, recall, specificity and confusion matrix. The proposed BTC-fCNN model outstrips the state-of-the-art and other well-known convolution neural networks (CNN).

## Introduction

Brain tumors are life-threatening having various types classified as benign and malignant. The malignant tumors have degree of malignancy that can be categorized into glioma, meningioma, and pituitary. For accurate and fast diagnosis, computer-aided diagnosis (CAD) systems become a must, especially with the advancement of deep learning networks that attract scientists to implement them for supporting healthcare [1–3]. From the clinical perspectives, the improvements in the image enhancement, object detection, and image classification pulled the consideration for early disease diagnosis and treatment plans [4–6]. To provide various views of tissues and organs, such as the brain, the magnetic resonance imaging (MRI) has been effectively enacted to analyse, monitor, diagnose and treat brain tumors.

For efficient diagnosis, several methods for brain tumor classification based on MRI images have been conducted. For example, Cheng et al*.* [7] implemented brain tumor classification for glioma, pituitary, and meningioma, using Gray level co-occurrence matrix (GLCM), and bag-of-words (BoW) model. The results reported 91.28% classification accuracy using BOW model. Furthermore, Ismael et al*.* [8] classified same three brain tumor types by combining statistical features and neural network, which achieved 91.9% classification accuracy. Ari et al*.* [9] classified the benign and malignant tumors using local smoothing, and nonlocal means procedures to remove noise, then applying the extreme learning machine local receptive fields (ELM-LRF). The results depicted 97.18% classification accuracy, 97.12% specificity, and 96.80% sensitivity. Gumaei et al. [10] established brain tumor classification depending on a hybrid feature extraction using principal component analysis (PCA) with normalized descriptors, followed by regularized extreme learning machine achieving 94.23% accuracy.

In contrast, different deep learning-based models were implemented for brain tumor classification, for example, Sajjad et al*.* [11] implemented brain tumor classification using pre-trained convolution neural network (CNN) with data augmentation based on VGG-19. This model was fine-tuned to provide 94.58% classification accuracy, 88.41% sensitivity, and 96.12% specificity. Kutlu et al*.* [12] established a classification model based on AlexNet CNN with 10 layers using different trainable parameters on 300 images, namely 100 glioma, 100 meningioma, and 100 pituitary tumors. Moreover, a pre-trained VGG19 model was proposed by Swati et al*.* [13] for brain tumor classification of the same brain tumor types achieving a mean accuracy of 94.82%. Excitation and squeeze ResNet model were implemented by Ghosal et al*.* [14] for brain tumor classification with 89.93%, and 93.83% accuracies without and data augmentation, respectively. Anaraki et al*.* [15] proposed a classification model based on the structure of the CNN, which consisted of convolution layers, max-pooling layers, and a fully connected layer with genetic algorithm. The results demonstrated accuracy of 94.2% on classifying the three tumor types with 90.9% accuracy for classifying three grades of Glioma. Deepak et al*.* [16] introduced a pre-trained CNN network with transfer learning to classify the three classes of the brain tumor using a pre-trained GoogleNet leading to 97.1% accuracy.

Another trend based on combining two paths of the CNN was designed by Alshayeji et al*.* [17] for classification, which consisted of convolutional, dropout, max-pooling, batch normalization, flatten, and dense layers with applying Bayesian optimization. It reported an accuracy of 97.37%. Kakarla et al. [18]*.* exhibited brain tumor classification network based on CNN structure with eight layers, which achieved classification accuracy 97.42%, 97.41% precision, 97.42% recall, and 95.09% Jaccard. Kumar et al*.* [19] developed a classification network based on pre-trained ResNet-50 by replacing the output layer with average pooling and softmax layers for 97.08% classification accuracy with data augmentation, and 97.48% without augmentation. Table 1 summaries the previously mentioned techniques for brain tumor classification.

**Table 1:** Different classification techniques for brain tumor diagnosis

Reference	Method	Number of images in the dataset	Limitations	Accuracy %
Cheng et al*.* [7]	BoW, intensity histogram and GLCM	3064	Elevated computational complicatedness	91.28
	Ring from partition for classification			
Ismael et al*.* [8]	The histogram and the GLCM for feature extraction	3064	Elevated computational complicatedness	91.9
	ANN for classification			
Ari et al*.* [9]	ELM-LRF for classification	108	Small dataset	97.18
			Inappropriate for another training dataset	
	Watershed segmentation for segmentation			
Gumaei et al. [10]	(PCA) with GIST descriptors for feature extraction	3064	Elevated computational complicatedness	94.23
	Regularized extreme learning machine for classification			
Sajjad et al*.* [11]	VGG19 with data augmentation	3064	Elevated computational cost	94.58
			Large storage required	
Kutlu et al*.* [12]	Based on AlexNet	300	Small dataset	98.6
			Elevated computational cost	
			Large storage requirements	
Swati et al*.* [13]	VGG with fine tuning	3064	Elevated computational cost	94.82
			Large storage requirements	
Ghosal et al*.* [14]	Based on AlexNet	3049	Elevated computational cost	93.83
			Large storage requirements	
Anaraki et al*.* [15]	CNN with Genetic Algorithm	3064	Elevated computational cost	94.2
			Large storage requirements	
Deepak et al*.* [16]	GoogleNet with Transfer Learning	3064	Time-consuming	97.1
			Elevated computational cost	
			Large storage requirements	
Alshayeji et al*.* [17]	Aggregation of two paths from CNN	3064	Time-consuming	97.37
			Elevated computational cost	
			Large storage requirements	
Kakarla et al. [18]	Average pooling convolutional neural network	3064	Time-consuming	97.42
			Elevated computational cost	
			Large storage requirements	
Kumar et al*.* [19]	ResNet-50 with Global Average Pooling at the output layer	3064	Time-consuming	97.48
			Elevated computational cost	
			Large storage requirements	

Table 1 proved that using deep learning networks provided superior performance compared to the traditional classification methods. However, the limitations of the existing CNN-based classification networks include the increased number of layers for feature extraction leading to increased learning time requirements owing to the increased number of the learning parameters and complicated architecture. Also, these networks suffer from memory limitations due to enormous parameters. Since such limitations obstacle their use in real-time diagnostic systems, this paper exhibits automatic, accurate and fast multi-class classification system, which we named BTC-fCNN. The proposed system is accurately classify the three brain tumor types (i.e. pituitary, meningioma, and glioma tumors). It was inspired and designed to solve the limitations in the designed model by Kakarla et al. [18] for decreasing the large number of trainable parameters, reducing the computational cost/ learning time, and increasing the system performance. Thus, this paper proposed a fast, and efficient classification system BTC-fCNN for real-time brain tumor CAD system. The proposed model consists of 13 layers based on convolution layer with 3 × 3 kernel size, convolution layer with 1 × 1 kernel size, average pooling layer, fully connected layer, and softmax layer. The internal transfer learning during the cross-validation (retrained five-fold cross validation) was proposed. Consequently, the contributions of the proposed BTC-fCNN model can be summarized as follows:

1. Reducing the computational cost, number of parameters, and processing time for real-time diagnosis system relative to other well-known convolution neural networks and state-of-the-art methods by reducing the width, height, and the number of channels.
2. Achieving significant performance improvement using transfer learning, and retrained fivefold cross-validation.
3. Carrying out the advantage of the average pooling layer, which solves the problem of overfitting due to the unrequired optimization of the parameters [20].
4. Performing a high classification accuracy using the proposed network structure by applying the final proposed BTC-fCNN model using internal transfer learning between the successive folds during the fivefold cross-validation.
5. Studying different cases of the proposed model and comparative studies to ensure the superiority of the proposed BTC-fCNN model to differentiate between three brain tumor types: meningioma, glioma, and pituitary tumor.

The structure of the remaining sections in the paper is as follows. Section “Methodology” describes the proposed methodology, and the dataset in detail. Section “Experimental simulation results” discusses the result and the model evaluation. Section “Discussion” explicates the discussion, and comparison between the proposed model and the existing classification networks, followed by the conclusion exhibited in section “Conclusion”.

## Methodology

The proposed brain tumor classification system BTC-fCNN is proposed for efficient performance with good computational cost, and time reduction. The proposed model involves different layers, which are studied in three cases, initial proposed model (Case 1, and Case 2) using the proposed new structure, then finally we concluded the final proposed model in Case 3. In these cases, the fivefold cross- validation without transfer learning was applied in case 1 as an initial proposed model, then, five iterations with transfer learning was applied using fivefold cross-validation in case 2 of the initial proposed model. Finally, case 3 included the final concluded proposed model, which was based on applying the transfer learning internally between the different successive folds during the fivefold cross-validation.

### Brain Tumor Dataset

**Fig. 1:**
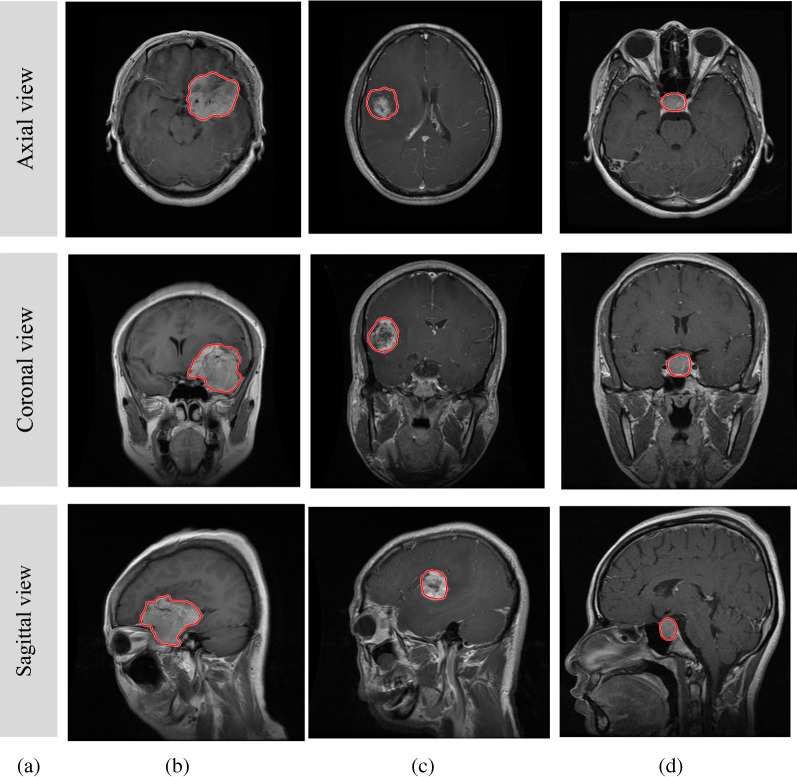
The three types of brain tumor with different views, where **a** capturing view, **b** Meningioma tumor type, **c** Glioma tumor type, and **d** Pituitary tumor type

In the proposed model’s cases, the 2D MRI images from a freely available dataset [21] were used to evaluate the proposed brain tumor classification networks. This dataset comprises of 3064 contrast-enhanced T1-weighted MR images, which were collected from 233 patients at three views, namely coronal, sagittal, and axial views. It includes three brain tumor types specifically 708 slices for meningioma, 1426 slices for glioma, and 930 slices for pituitary tumor. Figure 1 illustrated sample of the used dataset.

### Traditional Convolution Neural Network

For a large number of images, the complexity and the computational time increase. The CNN network is used as the images are vectorized in the simple format by observing there features, where the CNN architecture consists of multiple layers, including input layer, convolution layer, fully connected layer, and the output layer. In our proposed model, the input MRI images are resized to 256 × 256 × 1, which refer to width, height, and channel number for images. Then, the convolution layer is represented to capture the low-level features, whereby increasing the number of layers provides high-level features from the input images. These features consist of color, edges, and gradient orientation. The convolution layer consists of a set of convolution kernels, called filters, which convolved with the input to produce the output features. At the initial training of the network, the initial kernel weights are initiated with random values, then, at each epoch in the training phase, the weights are adjusted, and the kernel is learning to extract significant features. The discrete time representation for the convolution procedure, and the two-dimension case can be formulated as fallow [22]

1 $$f_{C} (t) = (y * k)(t) = \sum\limits_{b = - \infty }^{\infty } {y(b)k(t - b)}$$

2 $$f_{C} (i,j) = (X * W)(i,j) = \sum\limits_{n} {\sum\limits_{m} {X(i,j) * W(i - n,j - m)} }$$

where $f_{C} (t)$ and $f_{C} (i,j)$ are the convolution operations for the case of the single input y, and the two-dimension input *X*, respectively. Also, $k$ and $b$ are the kernel filter and the time shifting, respectively, in addition, $i$ and $j$ describe the desired matrix area requisite after applying the convolution procedure.

For any convolution layer, the fully connected (FC) layer, and the non-linear activation functions are employed to allow the learning of the network from more complicated features and to allow nonlinear mapping of inputs to outputs. The Rectified Linear Units (ReLU) function is the most widely used activation function to improve the training time and overcome the problem of the vanishing gradient. It requires the minimum computational load in comparison to other functions. The activation functions, namely ReLU function $g_{1} (x),$ sigmoid function $g_{2} (x)$ and tanh function $g_{3} (x)$ as follow [23]

3 $$g_{1} (x) = \left\{ {\begin{array}{*{20}c} {0,x < 0} \\ {x,x \ge 0} \\ \end{array} } \right.$$

4 $$g_{2} (x) = \frac{1}{{1 + e^{ - x} }}$$

5 $$g_{3} (x) = \frac{2}{{1 + e^{ - 2x} }}$$

For the one convolution layer, if the input size $a^{{\left[ {L - 1} \right]}}$ of this layer has $h^{{\left[ {L - 1} \right]}} \times w^{{\left[ {L - 1} \right]}}$ dimension, and $n^{{\left[ {L - 1} \right]}}$ channels, the forward pass process of the convolution layer and resultant feature size $a^{\left[ L \right]}$ from convolution procedure can described as [24]

6 $$X^{\left[ L \right]} = W^{\left[ L \right]} a^{{\left[ {L - 1} \right]}} + b^{\left[ L \right]}$$

7 $$a^{\left[ L \right]} = g\left( {X^{\left[ L \right]} } \right)$$

8 $$h^{\left[ L \right]} = \left\lfloor {\frac{{h^{{\left[ {L - 1} \right]}} - f^{\left[ L \right]} + 2P^{\left[ L \right]} }}{{s^{\left[ L \right]} }} + 1} \right\rfloor$$

9 $$w^{\left[ L \right]} = \left\lfloor {\frac{{w^{{\left[ {L - 1} \right]}} - f^{\left[ L \right]} + 2P^{\left[ L \right]} }}{{s^{\left[ L \right]} }} + 1} \right\rfloor$$

where $$W^{\left[ L \right]}$$, and $$b^{\left[ L \right]}$$ are the weight and bias of the convolution layer, also, $$h^{\left[ L \right]}$$ and $$w^{\left[ L \right]}$$ indicate the height and weight of the resultant features map, respectively. The size of the filter, the padding of convolution process, and the stride of the convolution process are represented by $$f^{\left[ L \right]} ,$$$$p^{\left[ L \right]}$$ and $$s^{\left[ L \right]}$$, respectively. The computational cost ($$C$$) of the CNN network analyzes the performance of the network, which is computed for each convolution layer as:

10 $$C = f^{\left[ L \right]} \times f^{\left[ L \right]} \times n^{{\left[ {L - 1} \right]}} \times h^{\left[ L \right]} \times w^{\left[ L \right]} \times n_{f}$$

where $f^{\left[ L \right]}$ × $f^{\left[ L \right]}$ is the kernel size, $n^{{\left[ {L - 1} \right]}}$ indicates the number of the input channel, $n_{f}$ is the number of filters, and $h^{\left[ L \right]} ,w^{\left[ L \right]}$ refer to the height and weight of the output of the layer, respectively.

After the convolution layer, the average pooling layer takes the feature maps of larger size and reduces them to maps of smaller size to sub-sample the feature vector for shrinking the width and the height of the feature map. It retains the most dominant information within each step of the pool, while reducing the feature map size, where [25]

11 $$w_{p}^{[l]} = \frac{{w^{{\left[ {l - 1} \right]}} - f^{[l]} }}{{s^{[l]} + 1}}$$

12 $$h_{p}^{[l]} = \frac{{h^{{\left[ {l - 1} \right]}} - f^{[l]} }}{{s^{[l]} + 1}}$$

Here, $w_{p}^{[l]}$ and $h_{p}^{[l]}$ are the width and height of the output from the pooling layer, respectively, $s^{[l]}$ is the step number, and $f^{[l]}$ is the size of the filter.

The final layer in the CNN network is the classification layer, which comprises of the flatten layer, the fully connected layer and Softmax layer. The flatten layer is responsible for the conversion of 2D matrix format to a single-column vector. The fully connected layer responsible for computing the size of the volume relative to the class score. It predicts the label class by interpreting the vectorized input features. Then, the softmax layer is used in the multi-class classification to take the predicted scores as the input and produce the output in the range 0 to 1 representing the probability of the class. The decision output of classification taken depending on which class has high probability value, which is given by [26]:

13 $$P(Y = j\left| {X,W,b)} \right. = \frac{{e^{{X^{T} Wj}} }}{{\sum\limits_{j = 1}^{m} {e^{{X^{T} Wj}} } }}$$

where $W$ and $b$ are the vectors of weights. For multi-class classification, the cross entropy loss is computed by comparing the predicted and true labels. However, the three classes encoded with an integer numbers from 0 to 2 instead of one-hot encoding. Sparse categorical cross-entropy loss function is especial case of cross entropy, which is suitable for the deep networks as the cross entropy depends only on the neuron output instead of the gradient of the activation function. Consequently, the vanishing gradient problem was mitigated between the layers. The loss function can be formulated as follows [27]

14 $$L = - \sum\limits_{c = 1}^{m} {y_{o,c} \log (p_{o,c} )}$$

where $y$ is binary indicator (1 or 0) if the label of the class $c$ is the correct classification for the observation $o$, and $p$ is the predicted probability as the observation $o$ is of the class $c$.

### One by One Convolution Layer

In the proposed model, a 1 × 1 convolution layer [20] is used, which is an especial case of the convolution layer, which has 1 × 1 kernel. It is employed to overcome the drawback of the high computational cost of the convolution layer. This layer is used before every convolution layer to shrink the channel number, consequently, reducing the computational cost. For example, if the (256, 256, 32) input (from previous layer) is applied to the convolution layer with 3 × 3 kernel, and 32 filters, the output dimension will be (254, 254, 32), and computational cost of this layer is 3 × 3 × 32 × 254 × 254 × 32 = 594,579,456. Nonetheless, in the case of using 1 × 1 convolution layer of 10 filters before the convolution layer, the computational cost will be achieved 1 × 1 × 32 × 254 × 254 × 10 + 3 × 3 × 10 × 254 × 254 × 32 = 206,451,200 only. Consequently, the 1 × 1 convolution layer is used for computational cost reduction, which is used in the proposed system.

### Transfer Learning

The transfer learning is mainly depends on the obtained knowledge from the previously trained model to learn the dataset [28] for enhancing the learning in a target domain by employing the knowledge in the source domain and the learning task. It is defined as inductive transfer learning upon the availability of labelled data in the source and target domains of classification tasks. The domain can be represented as [29]:

15 $$D = \left( {v_{i} l_{i} } \right)\forall i$$

where $v_{i}$ and $l_{i}$ are the feature vector, and the class label for the ith sample of training, respectively.

### Proposed CNN- Based Models for Multi-Class Classification

**Fig. 2:**
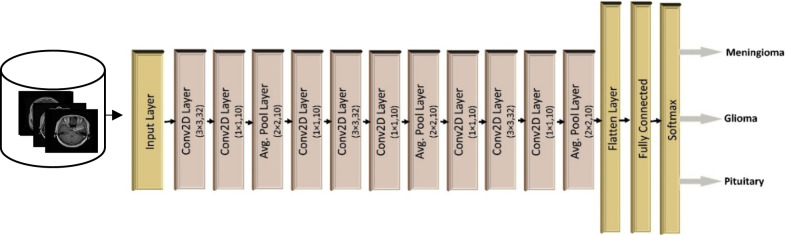
The block diagram of the proposed model’s general framework

In our proposed BTC-fCNN models, we take the benefits of using the 1 × 1 convolution layer as well as proposing an internal transfer learning procedure within the folds of the cross-valiadation to realize fast classification process with high accuracy. The transfer learning is employed for accurate and rapid train of the CNN, whereby the weights of CNN are not initiated from scratch [28]. This is definitely different from the training process on the traditional machine, where the input data learns from the start and need long learning time. The main strategy of the transfer learning is to save the parameters, and train the model to be used again in the same or other applications. Thus, the proposed model consists of 13 layers with involoving of covulation layer of 3 × 3 kernel and ReLU activation function, 1 × 1 convolution layer with ReLU activation function, average pooling with 2 × 2 kernel size, fully connected layer, and the softmax layer as exhibited in Fig. 2.

The general framework of the proposed model was studied in the initial proposed model’s case 1, and case 2, till we concluded the final proposed model in case 3, where (i) case 1: the five-fold cross-validation was applied on the general framework of the proposed model to evaluate the mean performance of the model over the five fold, (ii) case 2: five iterations with applying transfer learning between the iterations with five-fold cross validation was implemented, and (iii) case 3 (final concluded proposed model): the fivefold cross-validation was applied with retraining the model in each fold (internal transfer learning in the sequential fold).

#### The Initial Proposed Model (Case 1)

In the initial structure of the proposed model (case 1), fivefold cross-validation is used to provide the mean evaluation of the model in Fig. 2 over the five folds. In the training phase in each fold, the training images from the dataset with the corresponding class labels were resized into 256 × 256 × 1 and applied to train the model. The untrained model’s weights were initiated with a random weight value, then, the model was trained in each epoch for updating the weights using Adam optimizer, and sparse categorical cross-entropy loss function. In the testing phase in each fold, the trained proposed model was tested by the resized testing dataset to predict the classes of the brain tumor. Table 2 listed the parameters of the general framework of the proposed model.

**Table 2:** The configuration of the proposed model with the used parameters

Layer type	Number of filters	Kernel size	Output size	Number of parameter
Conv2D	32	3 × 3	(254, 254, 32)	320
Conv2D	10	1 × 1	(254, 254, 10)	330
AveragePooling2D	–	2 × 2	(127, 127, 10)	0
Conv2D	10	1 × 1	(127, 127, 10)	110
Conv2D	32	3 × 3	(125, 125, 32)	2912
Conv2D	10	1 × 1	(125, 125, 10)	330
AveragePooling2D	-	2 × 2	(62, 62, 10)	0
Conv2D	10	1 × 1	(62, 62, 10)	110
Conv2D	32	3 × 3	(60, 60, 32)	2912
Conv2D	10	1 × 1	(60, 60, 10)	330
AveragePooling2D	–	2 × 2	(30, 30, 10)	0
Flatten	–	–	9000	0
Dense	–	–	64	576,064
Softmax	–	–	3	195
Total parameters	583,613			
Trainable parameters	583,613			

#### The Initial Proposed Model (Case 2)

In the initial structure of the proposed model (case 2), five iterations using transfer learning are applied on the initial proposed model (in Fig. 2). The fivefold cross-validation was exploited, then, the resultant trained model was saved to retrain it in the next iteration which depicted in Algorithm 1.
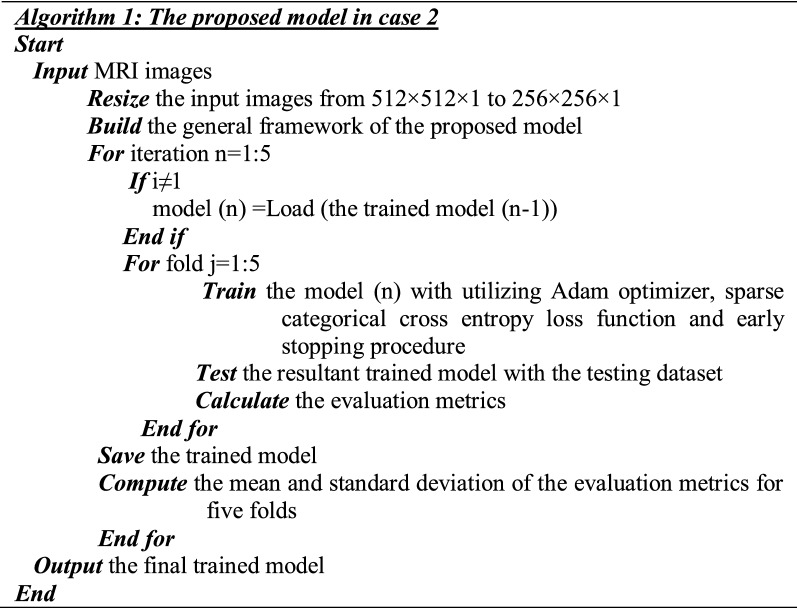


#### The Final Proposed BTC-fCNN Model (Case 3)

**Fig. 3:**
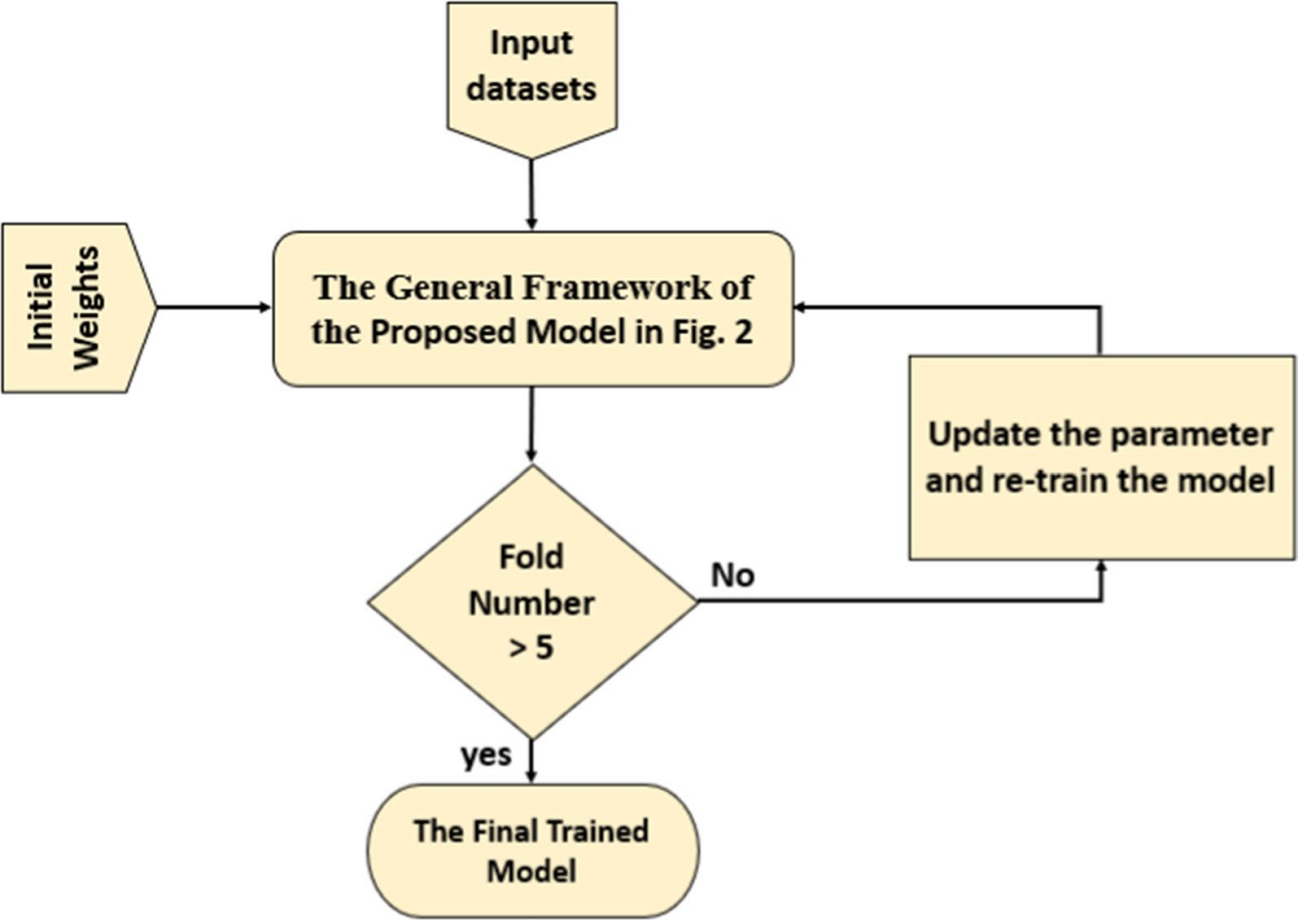
The final proposed BTC-fCNN model in case 3


In the final proposed BTC-fCNN model in case 3, Algorithm 2 is applied using fivefold cross-validation, but the trained model is stored in each fold, and retrained (internal transfer learning between the successive folds) in the next fold as described in Fig. 3.

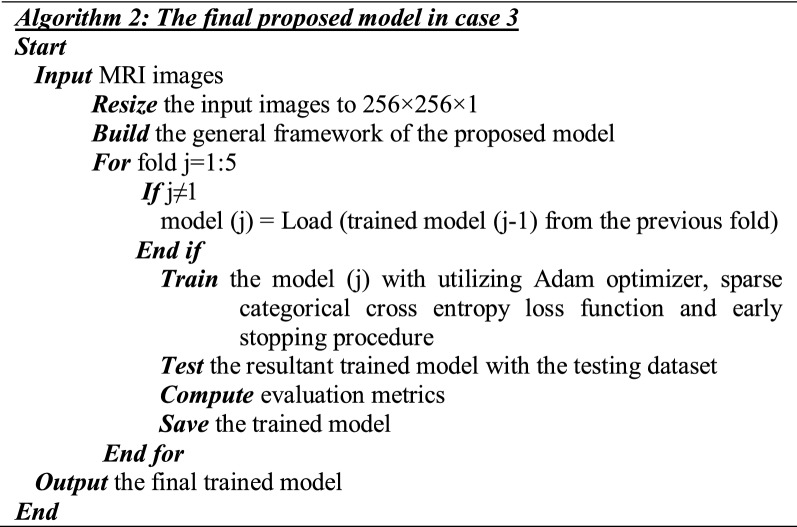


Figure 3 shows the proposed BTC-fCNN model using retrained five-fold cross-validation (internal transfer learning within the folds of the cross-validation). In the first fold, the general framework of the proposed model is trained from scratch with initial weights. Afterward, in the successive other folds, the transfer learning is applied in the other sequential folds to retrain the model in each fold until it reaches the final trained proposed BTC-fCNN model.

### Evaluation Metrics

To evaluate the different cases of the proposed BTC-fCNN model, different evaluation metrics were measured. These metrics include the confusion matrix, which is generated based on the predictions of the model, and the true labels. Moreover, the evaluation metrics, such as accuracy, losses, F1-score, precision, recall, specificity and time are calculated to compare between various models. These metrics are formulated as follows [30]:

16 $$Accuracy = \frac{TP + TN}{{TP + FP + FN + TN}}$$

17 $$Precison = \frac{TP}{{TP + FP}}$$

18 $$Recall = \frac{TP}{{TP + FN}}$$

19 $$F1 - score = \frac{2 \times recall \times precison}{{recall + precison}}$$

20 $$Specificity = \frac{TN}{{TN + FP}}$$

where $TN,TP,FP$ and $FN$ are the true negative, true positive, false positive, and false negative, respectively.

## Experimental Simulation Results

The proposed BTC-fCNN models were implemented using Python, and TensorFlow on the Google Colab. The used dataset in the present work is partitioned into 80% for training and validation, and 20% for testing. The different classes in the dataset were labeled into 0 meningioma, 1 glioma, and 2 pituitary. The applied hyper-parameters during the formation of the proposed models are 0.01 initial learning rate, and 10 epochs, and 25 batch size. The proposed models are employed Adam optimizer, and sparse categorical cross-entropy loss function with early stopping procedure. Furthermore, the proposed models were compared with well-known CNN, and state-of-the-art that were applied to the same dataset.

### The proposed model evaluation

The general framework of the proposed model is evaluated in case 1 and case 2 to reach the concluded final proposed BTC-fCNN model in case 3, where the output trained model from each fold is considered as the initial model in the next fold, which considered as retrained the model in each fold.

#### The results of the Initial of Proposed Model (Case 1)

**Fig. 4:**
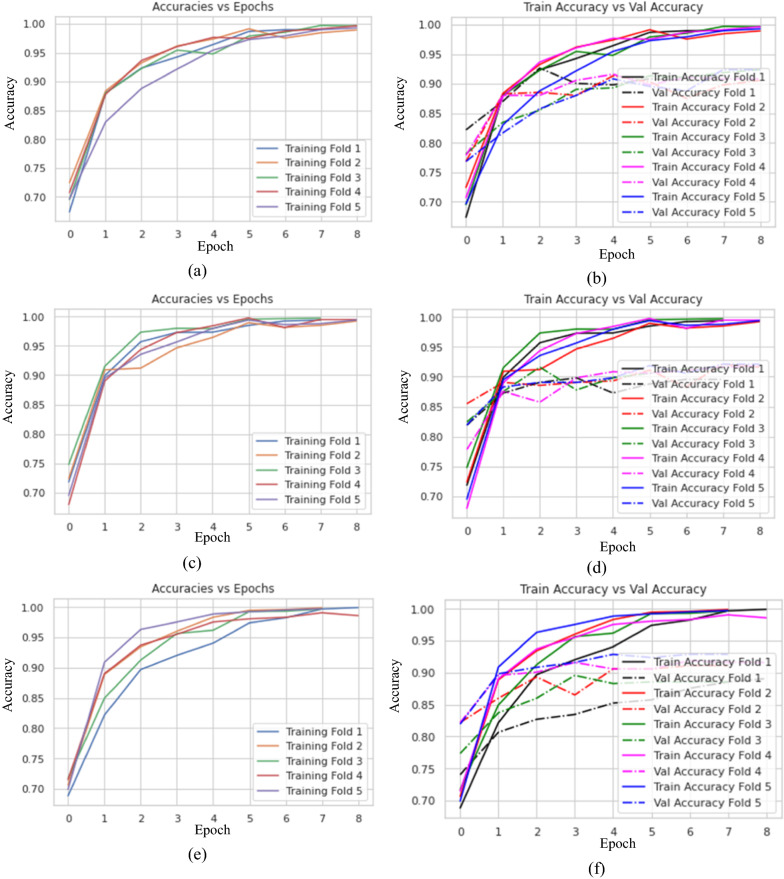
The training and validation accuracies with different folds and various numbers of filters, namely **a, b** 16 filters, **c, d** 10 filters, and **e, f** 8 filters

In this case, the model is evaluated when the fivefold cross-validation. Also, the model was studied at a different filter number in the convolution layer with 1 × 1 kernel size. Figure 4 displays the training and validation accuracy for different fold with various filter numbers, namely 16, 10 and 8 filters.

**Fig. 5:**
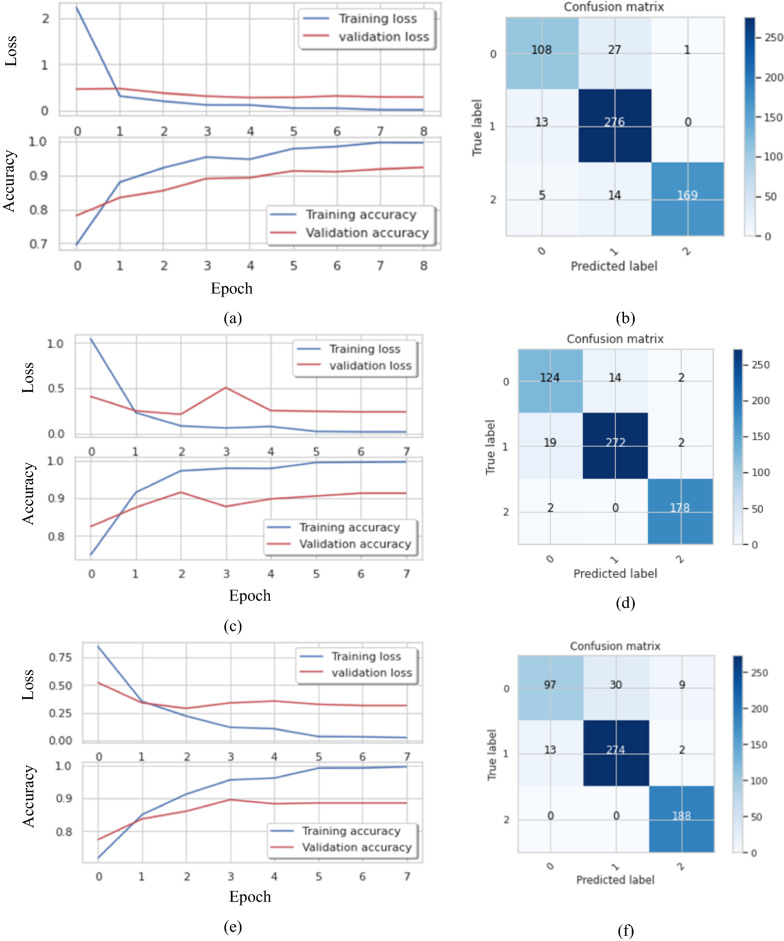
The training losses, validation losses and confusion matrix for different filters **a, b** 16 filters **c, d** 10 filters **e, f** 8 filters

In addition, Fig. 5 describes the accuracy, losses, and confusion matrix of the last fold five in the case of training, validation, and testing. Then, Table 3 reports the mean and standard deviation (SD) of the evaluation metrics for measuring the performance of the proposed model in case 1.

**Table 3:** The performance metrics of initial proposed model in case 1 using different number of filters

No. of filter	Accuracy %		Loss	F1-score %	Precision %	Recall %	Specificity %	Time (Sec.)	Number of model parameters
16 filters	Mean	90.96	0.28	90.02	90.18	89.97	95.27	49.43	933,587
	SD	± 1.55	± 0.04	± 1.73	± 1.65	± 1.87	± 0.86	± 6.51	
10 filters	Mean	93.08	0.24	92.21	92.47	92.01	96.34	41.43	583,613
	SD	± 0.44	± 0.03	± 0.64	± 0.58	± 0.86	± 0.29	± 1.61	
8 filters	Mean	91.25	0.29	90.13	90.72	89.85	95.34	37.56	466,987
	SD	± 1.81	± 0.07	± 2.09	± 2.13	± 2.16	± 0.89	± 3.68	

Table 3 clarifies that the proposed model with 10 filters accomplished the best performance compared to the other filter numbers. The mean accuracy of using 10 filters achieved 93.08% ± 0.44%, while the 16 and 8 filters achieved 90.96% ± 1.55%, and 91.25% ± 1.81%, respectively. The sub-model with 10 filters was carried out, which took 41.43 ± 1.61 s with a smaller number of trainable parameters 583.613. It described that the model performance was low due to train the model from scratch.

#### The Results of the Initial Proposed Model (Case 2)

The performance of the initial structure of proposed model in case 2 was investigated to compare it with the other models. This model consists of 5 iterations of transfer learning and applying fivefold cross-validation in each iteration. Tables 4, 5, 6, 7, 8 demonstrate the performance of the sub-model in each iteration.

**Table 4:** The performance evaluation of the initial proposed model in case 2 at iteration 1

Fold	Accuracy %	Loss	F1-score %	Precision %	Recall %	Specificity %	Time (Sec.)
1	92.82	0.24	91.75	92.39	91.26	96.11	42.33
2	93.64	0.21	93.11	92.81	93.43	96.75	39.53
3	92.82	0.26	92.15	92.39	91.92	96.19	42.88
4	92.66	0.28	91.49	91.61	91.41	96.08	40.78
5	93.46	0.22	92.52	93.15	92.03	96.56	40.64
Mean	93.08	0.24	92.21	92.47	92.01	96.34	41.43
SD	± 0.44	± 0.03	± 0.64	± 0.58	± 0.86	± 0.29	± 1.61

**Table 5:** The performance evaluation of the initial proposed model in case 2 at iteration 2

Fold	Accuracy %	Loss	F1-score %	Precision %	Recall %	Specificity %	Time (Sec.)
1	97.72	0.07	97.66	97.48	97.84	98.83	42.17
2	97.55	0.11	97.41	97.41	97.41	98.72	41.69
3	97.88	0.06	97.63	97.29	98.01	98.97	40.21
4	97.72	0.09	97.51	97.42	97.59	98.85	37.79
5	96.73	0.11	96.16	96.86	95.66	98.22	33.19
Mean	97.52	0.09	97.27	97.29	97.31	98.72	39.01
SD	± 0.46	± 0.02	± 0.63	± 0.25	± 0.95	± 0.29	± 3.67

**Table 6:** The performance evaluation of the initial proposed model in case 2 at iteration 3

Fold	Accuracy %	Loss	F1-score %	Precision %	Recall %	Specificity%	Time (Sec.)
1	99.02	0.03	98.82	98.74	98.89	99.55	41.71
2	98.21	0.05	98.03	97.79	98.27	99.14	41.68
3	98.53	0.05	98.42	98.54	98.31	99.23	40.31
4	98.53	0.06	98.28	98.29	98.28	99.28	41.65
5	98.69	0.04	98.58	98.68	98.49	99.29	41.39
Mean	98.59	0.05	98.43	98.41	98.45	99.29	41.35
SD	± 0.29	± 0.01	± 0.29	± 0.39	± 0.26	± 0.15	± 0.59

**Table 7:** The performance evaluation of the initial proposed model in case 2 at iteration 4

Fold	Accuracy %	Loss	F1-score %	Precision %	Recall %	Specificity%	Time (Sec.)
1	98.37	0.06	98.29	98.42	98.18	99.14	28.76
2	98.37	0.05	98.13	98.05	98.21	99.18	28.79
3	98.21	0.05	97.98	97.85	98.13	99.15	28.98
4	99.35	0.03	99.29	99.33	99.25	99.64	28.99
5	98.86	0.05	98.66	98.59	98.74	99.46	28.46
Mean	98.63	0.05	98.47	98.45	98.51	99.31	28.79
SD	± 0.47	± 0.01	± 0.52	± 0.57	± 0.49	± 0.23	± 0.22

**Table 8:** The performance evaluation of the initial proposed model in case 2 at iteration 5

Fold	Accuracy %	Loss	F1-score %	Precision %	Recall %	Specificity%	Time (Sec.)
1	98.21	0.06	97.99	98.13	97.88	99.07	29.09
2	99.18	0.04	99.05	98.88	99.23	99.62	28.51
3	98.37	0.05	98.05	98.14	97.98	99.19	28.69
4	99.02	0.03	98.95	98.91	98.98	99.51	28.67
5	98.37	0.05	98.28	98.19	98.38	99.18	28.91
Mean	98.63	0.05	98.46	98.45	98.49	99.31	28.77
SD	± 0.44	± 0.01	± 0.51	± 0.41	± 0.59	± 0.24	± 0.23

Tables 4, 5, 6, 7, 8 revealed that the mean accuracy and standard deviation for the five iterations were 93.08% ± 0.44%, 97.52% ± 0.46%, 98.59% ± 0.29%, 98.63% ± 0.47%, and 98.63% ± 0.44%, respectively, leading to the model stopping at the fourth iteration.

#### The Results of the Final Proposed BTC-fCNN Model (Case 3)

**Fig. 6:**
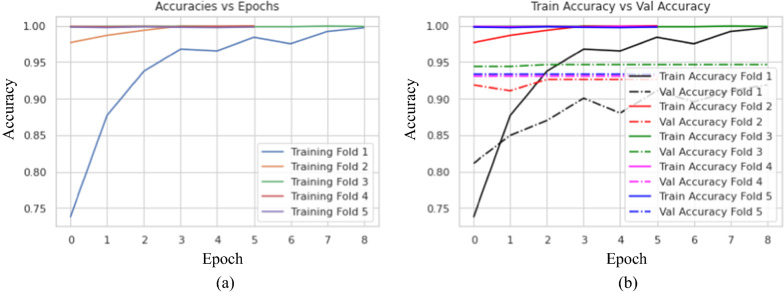
The accuracies of training and validation of the proposed BTC-fCNN model in case 3 **a** The training accuracy with various epochs, and **b** the training and validation accuracy with different epochs

The final proposed BTC-fCNN model was discussed using fivefold cross-validation with retraining the model in each fold. Figure 6 illustrates the proposed BTC-fCNN model accuracy of training and validation with each fold.

**Fig. 7:**
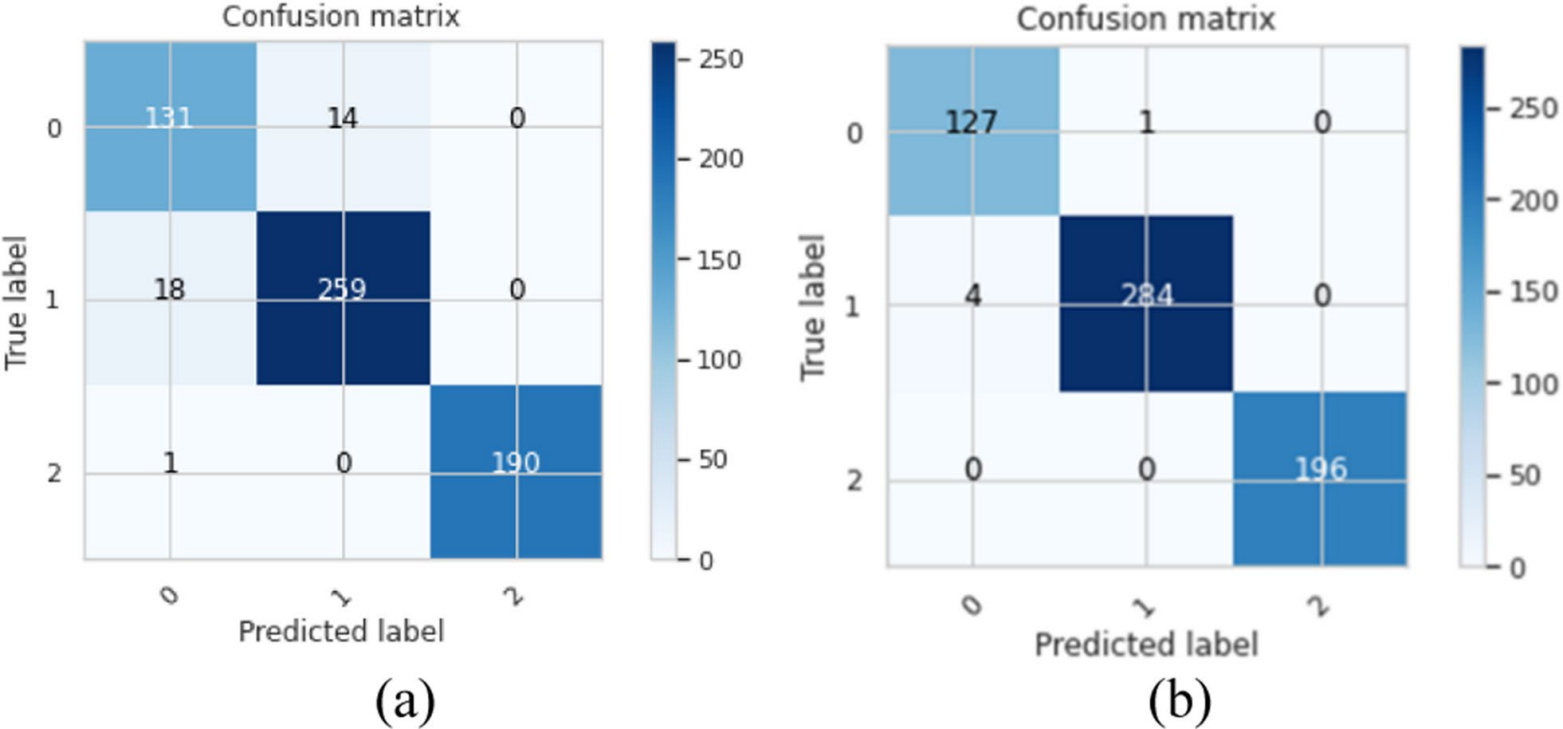
The confusion matrix for the proposed BTC-fCNN model in case 3, where **a** Fold 1, and **b** Fold 5

Figure 7 demonstrates the confusion matrix of the proposed BTC-fCNN model in the first and last fold. Furthermore, the BTC-fCNN model’s evaluation metrics are studied in Table 9.

**Table 9:** The performance evaluation metrics of the proposed BTC-fCNN model in case 3 with retraining in each fold

Fold	Accuracy %	Loss	F1-score %	Precision %	Recall %	Specificity%	Time (Sec.)
1	92.82	0.22	92.15	92.39	91.92	96.19	38.65
2	98.86	0.04	98.73	98.67	98.79	99.41	26.32
3	98.04	0.06	97.94	97.94	97.94	98.98	41.31
4	99.02	0.04	98.87	98.74	99.01	99.52	26.49
5	99.18	0.03	99.07	98.87	99.28	99.62	26.77

Table 9 exhibits that with increasing the number of folds, the accuracy of the model increased, and the time reduced. The accuracies of the model’s five folds were 92.82%, 98.86%, 98.04%, 99.02%, and 99.18%, respectively. To ensure that the model performance is stable, the fivefold cross validation was applied on the trained model from the fold 4. Table 10 clarifies the fivefold cross validation on the proposed BTC-fCNN model after fold 4.

**Table 10:** The proposed BTC-fCNN model performance in case 3 after applying five-fold on the trained model of fold 4

Fold	Accuracy%	Loss	F1-score %	Precision%	Recall%	Specificity%	Time (Sec.)
1	98.53	0.05	98.46	98.41	98.51	99.24	27.04
2	98.21	0.08	98.07	98.05	98.08	99.05	26.67
3	99.02	0.05	99.01	99.01	99.01	99.49	26.85
4	99.51	0.01	99.45	99.24	99.66	99.79	26.78
5	99.02	0.05	98.87	98.87	98.87	99.49	27.25
Mean	98.86	0.05	98.77	98.72	98.83	99.41	26.92
SD	± 0.45	± 0.02	± 0.53	± 0.48	± 0.59	± 0.28	± 0.23

**Fig. 8:**
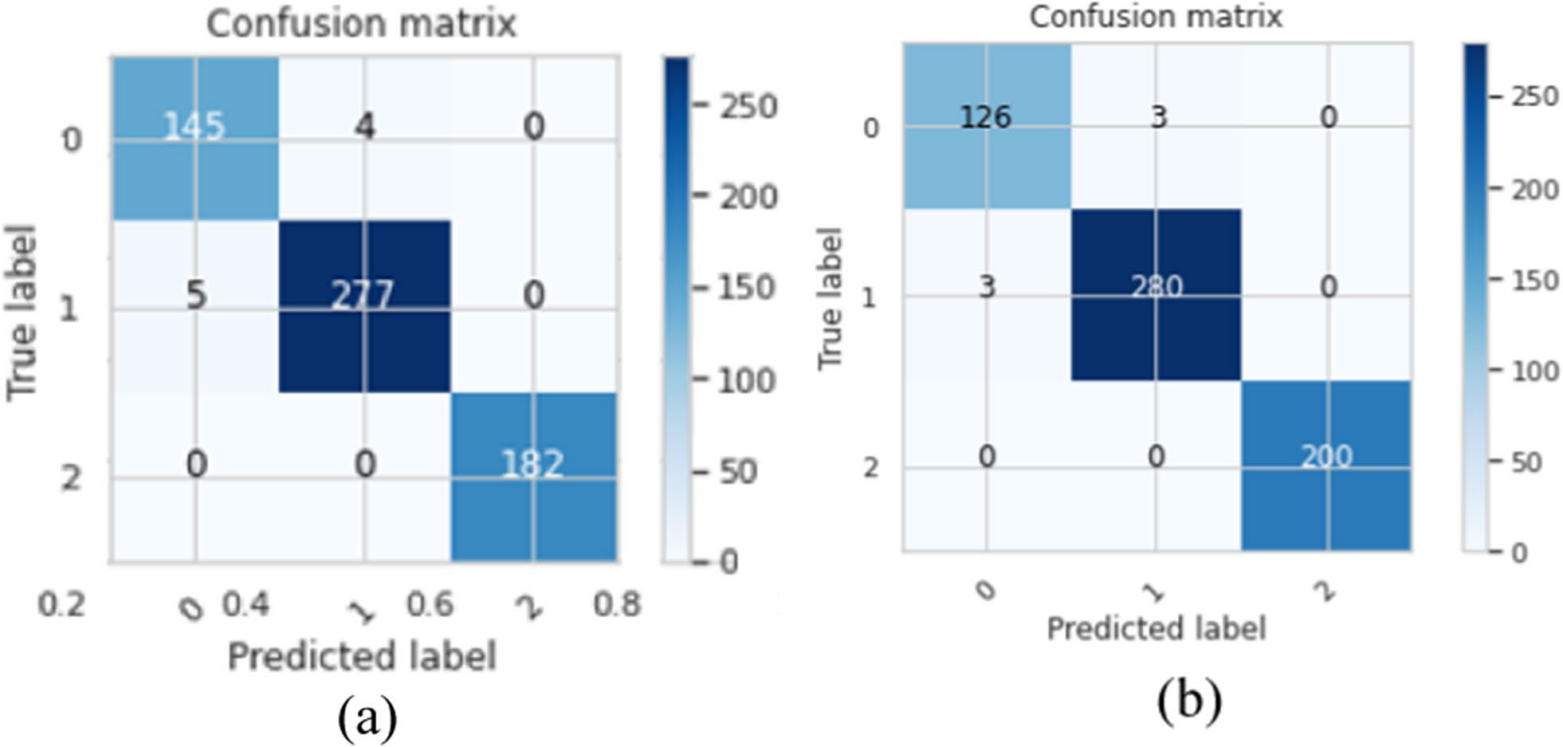
The confusion matrix of folds 1 and 5 after applying fivefold cross-validation on the proposed BTC-fCNN model in case 3 after fold 4, where **a** the confusion matrix of fold 1, and **b** the confusion matrix of fold 5

Table 10 indicates that the proposed BTC-fCNN model in case 3 achieved stable accuracy and losses with the different folds. The achieved mean accuracy of the model is 98.86% ± 0.45. Table 11 illustrates the first and the last fold performance with different classes. Also, Fig. 8 shows the confusion matrix in the first and the last fold.

**Table 11:** The performance of proposed multi-class classification model at the first and the last folds

Class	F1-score%	Precision%	Recall%	Specificity%	Accuracy %
Fold 1					
Meningioma	96.99	96.67	97.32	98.92	98.53
Glioma	98.41	98.58	98.23	98.79	
Pituitary	100	100	100	100	
Fold 5					
Meningioma	97.67	97.67	97.67	99.38	99.02
Glioma	98.94	98.93	98.94	99.09	
Pituitary	100	100	100	100

Table 11 demonstrates the performance of folds 1, and 5 in the proposed BTC-fCNN model (case 3) after applying the five-fold cross-validation, which retrained four folds with the different evaluation metrics for each class namely meningioma, glioma and pituitary tumor. This result indicates that the proposed BTC-fCNN model is accurately classified the pituitary class which obtained high performance compared to the meningioma and glioma classes.

The confusion matrix in Fig. 8a describes that using the first fold cross-validation leads to correctly classify 145 + 277 + 182 = 604 tumor images. Otherwise, the remaining 9 images of the tumor are erroneously classified, and the achieved classification accuracy equals $\frac{604}{{613}} \times 100 = 98.53\%$. Also, the confusion matrix of fold five in Fig. 8b exhibits that 126 + 280 + 200 = 606 images are successfully classified, and 6 images are wrongfully classified with classification accuracy equals $\frac{606}{{612}} \times 100 = 99.02\%$.

### Comparative Study of Proposed System Without 1 × 1 Convolution Layer

In this section, the proposed model without 1 × 1 convolution layer is evaluated using its proposed framework in Fig. 2.

#### Proposed Model Without Transfer Learning nor 1 × 1 Convolution Layer

**Fig. 9:**
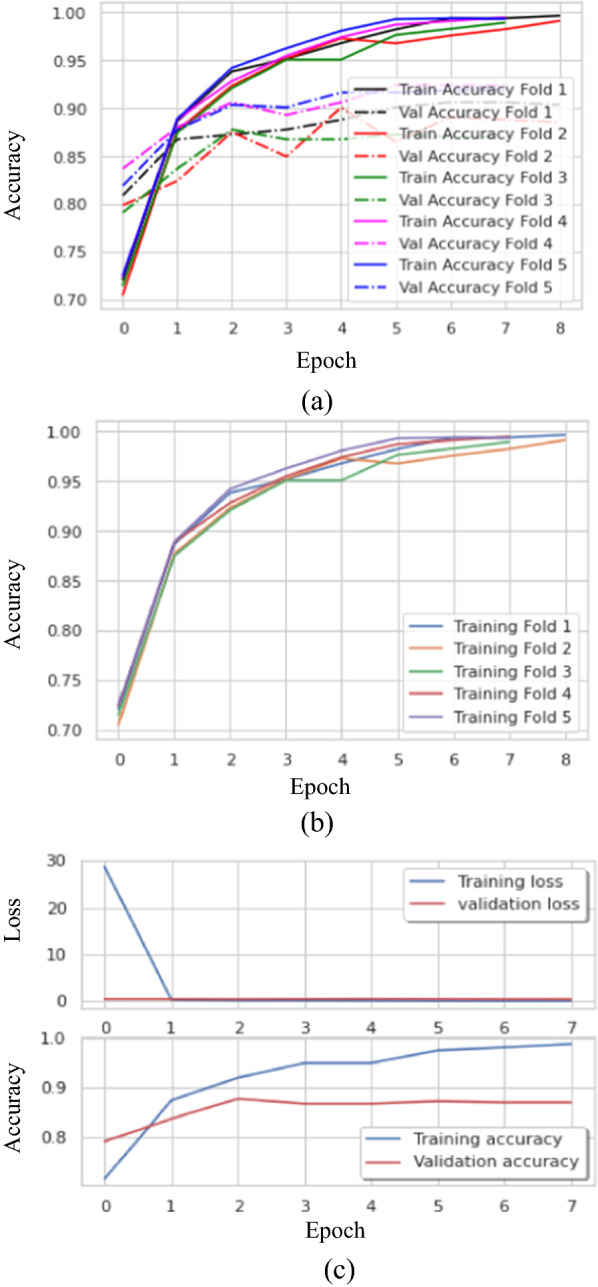
The accuracy and losses against the number of epochs of the proposed model without 1 × 1 convolution layer, where **a** training and validation for each fold, **b** training accuracy for different fold, and **c** an accuracy and losses for fold 5

In this section, the fivefold cross-validation was applied in the proposed model without 1 × 1 convolution layer to calculate its mean performance across the fivefold cross validations. Figure 9a, b describes the train, and validation accuracy in each fold during the ten epochs, but it may be stopped before the tenth epoch due to applying early stopping. Figure 9 (c) indicates the losses and accuracy for fifth fold of the proposed. In addition, Table 12 displays the model’s performance by calculating different metrics.

**Table 12:** The performance evaluation metrics of the proposed model without 1 × 1 convolution layer

Fold	Accuracy%	Loss	F1-score%	Precision%	Recall%	Specificity%	Time (sec)
1	90.54	0.29	89.83	91.08	89.19	94.66	66.75
2	92.33	0.20	91.58	91.41	91.78	96.14	82.53
3	90.21	0.33	89.16	88.66	89.78	95.07	82.39
4	92.66	0.34	91.96	92.26	91.77	96.22	82.35
5	92.81	0.25	91.37	91.61	91.15	96.15	82.62
Mean	91.71	0.28	90.78	91.01	90.73	95.65	79.33
SD	± 1.24	± 0.06	± 1.22	± 1.38	± 1.19	± 0.73	± 7.03

**Fig. 10:**
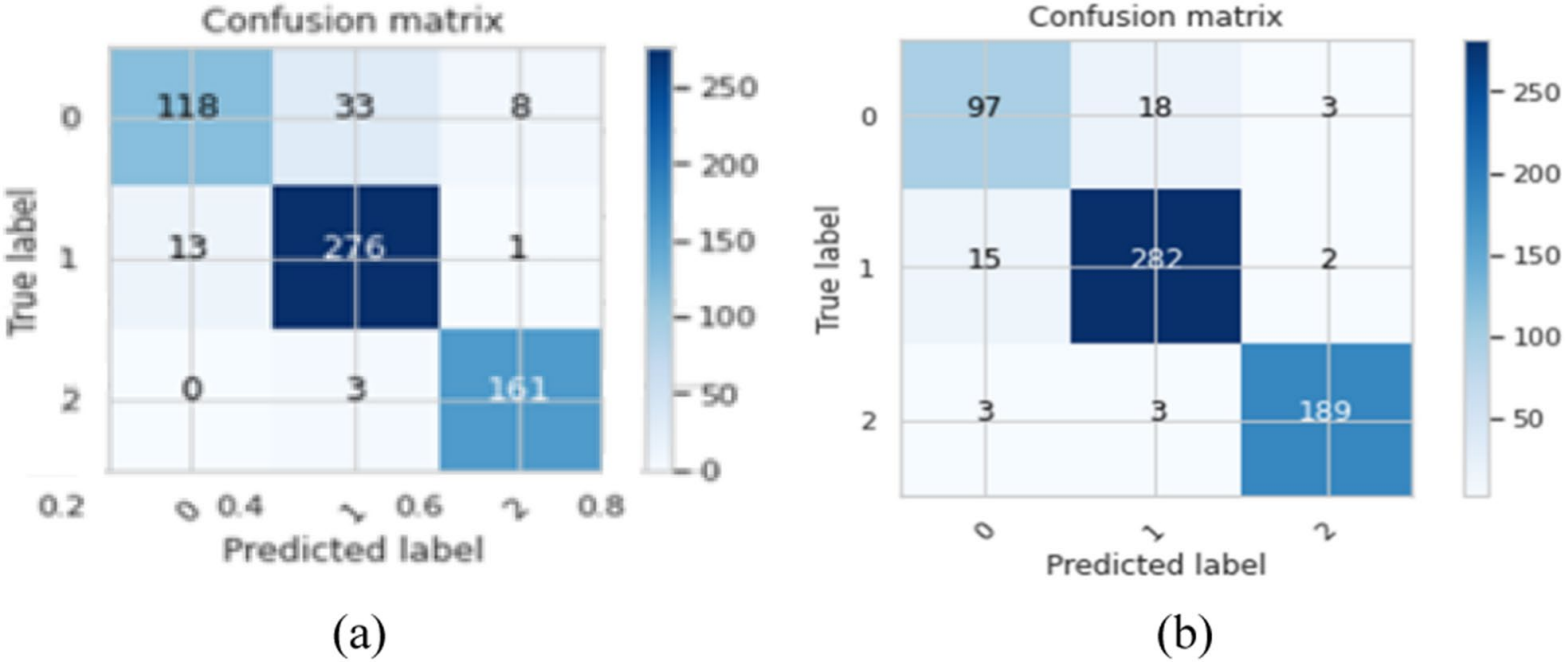
The confusion matrix for the first and last fold for the proposed model without 1 × 1 convolution layer, where **a** Fold 1, and **b** Fold 5

Figure 9 along with Table 12 reveal that the achieved accuracies are 90.54%, 92.33%, 90.21%, 92.66%, and 92.81% in fold 1, 2, 3, 4, and 5, respectively. The mean accuracy over the 5 folds is 91.71%, and ± 1.24%. Also, the mean processing time of the model for fivefold is 79.33 s. Figure 10a, b illustrates the confusion matrix of the model for the first and the last folds indicating that the proposed model without 1 × 1 convolution layer has poor performance.

#### Proposed Model with Transfer Learning and Without 1 × 1 Convolution Layer

In this section, the fivefold cross-validation with transfer learning on the 5-iterations is studied. In each iteration, the fivefold cross-validation is used, and then retrain the model in the next iteration. Tables 13, 14, 15, 16, 17 reported the performance of the proposed model at various iterations.

**Table 13:** The performance of the proposed model using transfer learning without 1 × 1 convolution layer at the first iteration

Fold	Accuracy%	Loss	F1-score%	Precision%	Recall%	Specificity%	Time (Sec.)
1	91.19	0.36	90.34	90.26	90.42	95.42	84.57
2	94.62	0.17	93.91	93.83	94.02	97.27	83.39
3	91.84	0.27	91.22	90.74	91.86	96.05	83.55
4	91.19	0.34	90.25	91.04	89.71	95.27	83.31
5	90.69	0.27	89.53	89.82	89.29	95.13	84.19
Mean	91.91	0.28	91.05	91.14	91.06	95.83	83.81
SD	± 1.57	± 0.07	± 1.71	± 1.58	± 1.92	± 0.88	± 0.55

**Table 14:** The performance of the proposed model using transfer learning without 1 × 1 convolution layer at the second iteration

Fold	Accuracy%	Loss	F1-score%	Precision%	Recall%	Specificity%	Time (Sec.)
1	95.27	0.13	94.64	94.34	95.01	97.66	82.59
2	96.41	0.13	96.09	96.31	95.93	98.14	60.11
3	95.92	0.13	95.66	95.69	95.64	97.87	61.97
4	95.11	0.12	94.49	94.45	94.55	97.42	60.62
5	97.22	0.11	96.79	96.82	96.79	98.61	82.64
Mean	95.99	0.12	95.53	95.52	95.58	97.94	69.59
SD	± 0.86	± 0.01	± 0.97	± 1.11	± 0.86	± 0.46	± 11.91

**Table 15:** The performance of the proposed model using transfer learning without 1 × 1 convolution layer at the third iteration

Fold	Accuracy%	Loss	F1-score%	Precision%	Recall%	Specificity%	Time (Sec.)
1	95.76	0.17	95.19	95.17	95.22	97.84	49.39
2	96.25	0.11	95.95	96.05	95.87	98.07	82.61
3	96.41	0.09	95.88	95.81	95.96	98.19	82.59
4	96.25	0.14	95.93	95.92	95.95	98.09	82.62
5	95.75	0.10	95.22	95.13	95.31	97.76	49.01
Mean	96.08	0.12	95.63	95.62	95.66	97.99	69.24
SD	± 0.31	± 0.03	± 0.39	± 0.43	± 0.37	± 0.18	± 18.29

**Table 16:** The performance of the proposed model using transfer learning without 1 × 1 convolution layer at the fourth iteration

Fold	Accuracy%	Loss	F1-score%	Precision%	Recall%	Specificity%	Time (Sec.)
1	97.23	0.08	97.05	97.05	97.05	98.56	82.59
2	97.39	0.13	97.13	97.21	97.06	98.64	60.76
3	97.23	0.05	96.88	96.57	97.22	98.69	82.62
4	97.06	0.07	96.77	96.94	96.61	98.42	60.29
5	99.02	0.03	98.86	98.71	99.01	99.54	56.39
Mean	97.59	0.07	97.34	97.29	97.39	98.77	68.53
SD	± 0.81	± 0.04	± 0.86	± 0.82	± 0.93	± 0.44	± 12.96

**Table 17:** The performance of the proposed model using transfer learning without 1 × 1 convolution layer at the fifth iteration

Fold	Accuracy%	Loss	F1-score%	Precision%	Recall%	Specificity%	Time (Sec.)
1	98.53	0.07	98.37	98.34	98.39	99.24	58.64
2	96.74	0.13	96.61	96.27	96.99	98.42	58.39
3	97.39	0.08	97.15	97.31	97.01	98.64	58.94
4	98.04	0.07	97.78	98.13	97.46	98.86	58.49
5	97.39	0.06	97.09	96.99	97.19	98.71	58.85
Mean	97.62	0.08	97.41	97.41	97.41	98.77	58.66
SD	± 0.69	± 0.03	± 0.68	± 0.85	± 0.58	± 0.31	± 0.23

Tables 13, 14, 15, 16, 17 depicted the superior performance of the proposed model using transfer learning and without 1 × 1 convolution layer with the increased number of iterations. However, after the fifth iteration, the model’s performance is unchangeable as it reached the stable state. The mean accuracy of the model in various iterations are 91.91% ± 1.57%, 95.99% ± 0.86%, 96.08% ± 0.31%, 97.59% ± 0.81%, and 97.62% ± 0.69% for iterations 1, 2, 3, 4, and 5, respectively. Additionally, the processing time in each iteration reached 83.81 s, 69.59 s, 69.24 s, 68.53 s and 58.66 s for iterations number 1, 2, 3, 4 and 5, respectively. It demonstrates that the model performance was enhanced due to applying transfer learning with the five iterations. Also, the mean accuracy in the fifth iteration achieved 97.62% ± 0.69%.

#### Proposed Model with Retrained Fivefold Cross Validation and Without 1 × 1 Convolution Layer

In this section, the fivefold cross validation utilized in the proposed model without 1 × 1 convolution layer, but with applying the transfer learning between the folds to improve the model performance by retraining the model in each fold. Table 18 elucidates the performance of the proposed model without 1 × 1 convolution layer after applying retrained model in each fold.

**Table 18:** The evaluation metrics of the proposed model without 1 × 1 convolution layer using retrained in each fold

Fold	Accuracy%	Loss	F1-score%	Precision%	Recall%	Specificity%	Time (Sec.)
1	91.19	0.06	90.16	89.68	90.72	95.59	85.12
2	97.88	0.06	97.65	97.82	97.65	98.87	86.25
3	98.85	0.04	98.74	98.76	98.72	99.39	80.31
4	98.53	0.06	98.48	98.55	98.43	99.24	69.68
5	98.03	0.05	97.73	97.47	97.73	99.07	65.34

Table 18 illustrates that the performance of the proposed model without 1 × 1 convolution layer is increased with retraining the model in each fold. The model accuracy achieved 91.19%, 97.88%, 98.85%, 98.53% and 98.03% for folds number 1, 2, 3, 4 and 5 respectively. To obtain the mean performance of the model and ensuring stability of the model, the fivefold cross validation applied on the trained model after fold 4 as exhibits in Table 19.

**Table 19:** The evaluation metrics of the proposed model without 1 × 1 convolution layer after applying five-fold on the trained model at fold 4

Fold	Accuracy%	Loss	F1-score%	Precision%	Recall%	Specificity%	Time (Sec.)
1	97.23	0.07	96.92	96.81	97.04	98.63	55.16
2	98.04	0.05	97.85	97.49	98.23	99.05	51.49
3	97.72	0.05	97.62	97.61	97.64	98.81	56.47
4	97.23	0.09	96.85	96.91	96.79	98.47	52.36
5	98.37	0.05	98.28	98.39	98.17	99.13	50.65
Mean	97.72	0.06	97.51	97.44	97.57	98.82	53.23
SD	± 0.51	± 0.02	± 0.61	± 0.63	± 0.65	± 0.28	± 2.48

Table 19 elucidates that the evaluation of the proposed model without 1 × 1 convolution layer using fivefold cross validation on the trained model of fold 4, which achieved mean accuracy of 97.72% ± 0.51%. Table 20 describes the comparative between the proposed model with and without the 1 × 1 convolution layer.

**Table 20:** The performance of the proposed model with and without using the 1 × 1 convolution layer

Model		Accuracy %	Loss	F1-score %	Precision %	Recall %	Specificity %	Time (Sec.)
The initial proposed model (case 1)	Mean	93.08	0.24	92.21	92.47	92.01	96.34	41.43
	SD	± 0.44	± 0.03	± 0.64	± 0.58	± 0.86	± 0.29	± 1.61
The initial proposed model (case 2)	Mean	98.63	0.05	98.46	98.45	98.49	99.31	28.77
	SD	± 0.44	± 0.01	± 0.51	± 0.41	± 0.59	± 0.24	± 0.23
The final proposed BTC-fCNN model (case 3)	Mean	98.86	0.05	98.77	98.72	98.83	99.41	26.92
	SD	± 0.45	± 0.02	± 0.53	± 0.48	± 0.59	± 0.28	± 0.23
Case 1 without 1 × 1conv. layer	Mean	91.71	0.28	90.78	91.01	90.73	95.65	79.33
	SD	± 1.24	± 0.06	± 1.22	± 1.38	± 1.19	± 0.73	± 7.03
Case 2 without 1 × 1conv. layer	Mean	97.62	0.08	97.41	97.41	97.41	98.77	58.66
	SD	± 0.69	± 0.03	± 0.68	± 0.85	± 0.58	± 0.31	± 0.23
Case 3 without 1 × 1conv. layer	Mean	97.72	0.06	97.51	97.44	97.57	98.82	53.23
	SD	± 0.51	± 0.02	± 0.61	± 0.63	± 0.65	± 0.28	± 2.48

Table 20 shows that the general framework model in various cases achieved the best result compared to using the model without 1 × 1 convolution layer. It achieved accuracies of 93.08%, 98.63%, and 98.86% in case 1, 2 and 3, respectively. However, the model without 1 × 1 convolution layer achieved low accuracy 91.71% in case 1, 97.62% in case 2, and 97.72%in case 3. Also, the proposed model had small trainable parameters and faster than the proposed model without 1 × 1 convolution layer.

### Comparative Study with Well-Known CNN Networks

The different cases of the proposed model are compared with the well-known CNN configurations. Various traditional pre-trained CNN models, such as VGG16 [31], VGG19 [31], InceptionV3 [32], ResNet50 [33], and MobileNet [34] were trained using the dataset of the three brain tumor classes to update their weights. Though the training phase of the traditional CNN using Adam optimizer and sparse categorical cross-entropy loss function with ten epochs, and 0.0001 learning rate. Table 21 interprets the comparative the proposed network and traditional CNN models.

**Table 21:** The comparative study between different well-known CNN and the proposed models

Model		Accuracy %	Time (Sec.)	Number of model parameters
VGG16	Mean	92.07	975.15	165,730,115
	SD	± 0.61	± 134.70	151,015,427
VGG19	Mean	93.05	1189.35	171,039,811
	SD	± 0.94	± 129.52	151,015,427
InceptionV3	Mean	80.35	1637.19	23,904,035
	SD	± 2.42	± 204.06	2,101,251
ResNet50	Mean	74.48	428.53	23,593,859
	SD	± 2.15	± 9.17	6,147
MobileNet	Mean	89.16	555.74	4,256,867
	SD	± 0.98	± 17.88	1,028,003
The initial proposed model in case 1	Mean	93.08	41.43	583,613
	SD	± 0.44	± 1.61	583,613
The initial proposed model in case 2	Mean	98.63	28.77	583,613
	SD	± 0.44	± 0.23	583,613
The final proposed BTC-fCNN model in case 3	Mean	98.86	26.92	583,613
	SD	± 0.45	± 0.23	583,613

Table 21 depicted that the proposed model in the different cases realized the best result compared to the traditional CNN. Besides, the proposed model in case 3, and case 2 achieved higher result compared to the proposed model in case 1. It is found that the proposed model in case 3 takes shorter time due to the early stopping procedure, where the proposed model in case 3 was trained and reached its steady state faster than the proposed model in case 2. The best CNN model accuracy is VGG19 network, which 93.05% ± 0.94, but it takes longer time of 1189.35 ± 129.52 s. On the other side, the proposed model in case 3 achieved 98.86% ± 0.45 accuracy in shorter time using less number of parameters.

## Discussion

The proposed BTC-fCNN model is also compared with the state-of-the-art operated on the same dataset, such as Sajjad et al*.* [11], Gumaei et al*.* [10], Anarki et al*.* [15], Swati et al*.* [13], Deepak et al*.* [16], Alshayeji et al*.* [17], Kakarla et al*.* [18], and Kumar et al*.* [19]. Table 22 reports the comparative study between the proposed models and the other state-of-the-art.

**Table 22:** Comparative study in terms of the accuracy with the state-of-the-art models

Reference	Model	Accuracy %
Gumaei et al. [10]	Regularized extreme learning machine	94.23
Sajjad et al*.* [11]	VGG19 with extensive data augmentation	94.58
Anarki et al*.* [15]	CNN with genetic algorithm	94.20
Swati et al*.* [13]	VGG19 with fine tuning	94.82
Deepak et al*.* [16]	GoogleNet with transfer learning	97.10
Alshayeji et al*.* [17]	Aggregation of two paths from CNN	97.37
Kakarla et al*.* [18]	Average pooling convolutional neural network	97.42
Kumar et al*.* [19]	ResNet-50 with Global Average Pooling at the output layer	97.48
The proposed BTC-fCNN model (case 3)	The proposed model with retraining the model in each fold during five folds	98.86

Table 22 proved that the proposed model’s cases are superior to the other models, where Gumaei et al*.* [10] achieved low accuracy 94.23% based on the hybrid feature extraction. *Sajjad *et al*.* [11] achieved 94.58% accuracy based on VGG-19 with 19 layers and large number of trainable parameters (171,039,811). Anaki et al*.* [15] attained the least accuracy of 94.20% using CNN structure without transfer learning. The VGG19 network with 19 layers and large number of 171,039,811 trainable parameters introduced by Swati et al*.* [13] accomplished 94.82% accuracy. Deepak et al*.* [16] extracted features using GoogleNet using many layers and parameters. Also, the CNN network structure with a large number of parameters was designed by Alshayeji et al*.* [17] and Kakarla et al*.* [18]. In addtion, Kumar et al*.* [19] accomplished 97.48% accuracy using ReNet-50 with 50 layers and a large number of 23,593,859 trainable parameters, while the proposed model achieved the best performance with small number of parameters. The final proposed BTC-fCNN model (case 3) achieved 98.86% accuracy with a small number of 583,613 trainable parameter only, while the existing approaches accuracy accomplished different accuracy values ranging from 94.20% to 97.48%.

## Conclusion

The classification of different classes developed as a complicated task for automation of brain tumor diagnosis. This paper introduced a proposed BTC-fCNN model to overcome the drawbacks of the existing CNN networks, which suffer from high computational cost and learning time. The proposed BTC-fCNN model consists of 13 layers with 583,613 trainable parameters, namely convolution layer with 3 × 3 kernel size, 1 × 1 convolution layer, average pooling layer, fully connected layer, and softmax layer. It was applied on 3064 MRI images from the Figshare dataset for classifying three classes, including glioma, pituitary, and meningioma tumor. Five iterations were used with transfer learning and fivefold cross validation (the initial structure of the proposed model in case 2), and retrained model in five-fold cross validation (the final proposed BTC-fCNN model “case 3”).

The proposed model achieved high accuracy compared to existing networks of 98.63% and 98.86% for the initial structure of the proposed model in case 2 and the final proposed BTC-fCNN model in case 3, respectively. Conversely, the state-of-the-art models achieved accuracy values ranging from 94.20% to 97.48%. Also, the final proposed BTC-fCNN model in case 3 achieved the shortest learning time compared to the other cases in the present study. Moreover, it attained at the shortest learning time with a computational cost reduction as it had small number of trainable parameters. In the future work, we will apply the proposed model in other different diseases such as classification for ECG beat [35] and diabetic eye diseases [36].

## Data Availability

The dataset used to evaluate the proposed system and support the findings of this study are available in the following hyperlink to dataset https://figshare.com/articles/dataset/brain_tumor_dataset/1512427/ that has the following DOI 10.6084/m9.figshare.1512427.v5.
